# The prognostic and therapeutic implications of distinct patterns of argininosuccinate synthase 1 (ASS1) and arginase-2 (ARG2) expression by cancer cells and tumor stroma in non-small-cell lung cancer

**DOI:** 10.1186/s40170-021-00264-7

**Published:** 2021-08-03

**Authors:** Alexandra Giatromanolaki, Adrian L. Harris, Michael I. Koukourakis

**Affiliations:** 1 Department of Pathology, University Hospital of Alexandroupolis, Democritus University of Thrace, PO BOX 12, 68100 Alexandroupolis, Greece; 2 Department of Radiotherapy/Oncology, University Hospital of Alexandroupolis, Democritus University of Thrace, PO BOX 12, 68100 Alexandroupolis, Greece; 3 Cancer Research UK, Molecular Oncology Laboratories, Weatherall Institute of Molecular Medicine, University of Oxford, Oxford, UK

**Keywords:** Arginine, Arginase, ARG2, Argininosuccinate synthase, ASS1, Angiogenesis, TILs, Lung cancer

## Abstract

**Background:**

Arginine (Arg) is essential for cancer cell growth and also for the activation of T cells. Thus, therapies aiming to reduce Arg utilization by cancer may prove detrimental for the immune response.

**Methods:**

We examined the expression of two major enzymes involved in arginine depletion and replenishment, namely arginase ARG2 and argininosuccinate synthase ASS1, respectively, in a series of 98 NSCLCs. Their association with immune infiltrates and the postoperative outcome were also studied.

**Results:**

ARG2 was expressed mainly by cancer-associated fibroblasts (CAFs) (58/98 cases; 59.2%), while ASS1 by cancer cells (75/98 cases; 76.5%). ASS1 and ARG2 expression patterns were not related to hypoxia markers. Auxotrophy, implied by the lack of expression of ASS1 in cancer cells, was associated with high angiogenesis (*p* < 0.02). ASS1 expression by cancer cells was associated with a high density of iNOS-expressing tumor-infiltrating lymphocytes (^iNOS+^TILs). ARG2 expression by CAFs was inversely related to the TIL-density and linked with poorer prognosis (*p* = 0.02). Patients with ASS1 expression by cancer cells had a better prognosis especially when CAFs did not express ARG2 (*p* = 0.004).

**Conclusions:**

ARG2 and ASS1 enzymes are extensively expressed in NSCLC stroma and cancer cells, respectively. Auxotrophic tumors have a poor prognosis, potentially by utilizing Arg, thus reducing Arg-dependent TIL anti-tumor activity. ASS1 expression in cancer cells would allow Arg fueling of ^iNOS+^TILs and enhance anti-tumor immunity. However, upregulation of ARG2 in CAFs may divert Arg from TILs, allowing immune escape. Identification of these three distinct phenotypes may be useful in the individualization of Arg-targeting therapies and immunotherapy.

**Supplementary Information:**

The online version contains supplementary material available at 10.1186/s40170-021-00264-7.

## Background

l-arginine (Arg) is a conditionally essential amino acid that plays an important role in energy metabolism and signaling pathways in many tissues, such as muscles and nerves [1]. Under stressful conditions, e.g., surgery or trauma, supplementation of dietary intake is necessary to provide the increased demand for Arg. It also has an important role in the secretion of growth hormones and immune function. Arg is a precursor for nitric oxide NO, urea, ornithine, and agmantine. Arg deprivation in normal cells leads to cell cycle arrest and quiescence, while cancer cells may continue their growth and activate apoptosis [2]. Moreover, deprivation of Arg inhibits the mTOR pathway and triggers autophagic cell death pathways [3].

Although normal cells can synthesize Arg from citrulline through the activity of argininosuccinate synthase (ASS1), this latter enzyme is downregulated in many tumors, making them auxotrophic [4]. This observation led to the hypothesis that targeting Arg-degrading enzymes could be of anti-tumor therapeutic value [5]. Four major enzymes are involved in arginine catabolism [6]. Arginine deiminase (ADI) catalyzes the irreversible deimination of Arg and its transformation to citrulline. Arginine decarboxylase (ADC) metabolizes Arg to agmatine. Nitric oxide synthases (neuronal, endothelial, and inducible NOS) catalyze the production of NO and citrulline from Arg. Finally, arginases (ARG) convert Arg to ornithine and urea. Arginases exist in two distinct forms: the type I (ARG1) that is cytosolic and is mainly expressed by hepatocytes, and the type II (ARG2) that is located in the mitochondrial matrix and is expressed in many tissues [6].

In the new era of cancer immunotherapy, Arg and enzymes related to its metabolism have been investigated. Arg is essential for T-lymphocytes as it supports the expression of T cell receptors (TCR) by regulating the translation of the ζ-chain peptide, an important component of the TCR-complex (together with CD3), and the subsequent activation of CD4 and CD8 T cells [7, 8]. Arg utilization by T cells is minimal under resting conditions and increases sharply after T cell activation. T cells have the ability to synthesize arginine from citrulline by up-regulating ASS1 [9]. As arginine auxotrophy (lack of expression of ASS1 by cancer cells) and exogenous Arg consumption widely characterize tumors, it is postulated that Arg-depleted tumor microenvironment is a critical condition preventing the thriving of tumor-infiltrating T cells and promoting cancer escape from immune surveillance [10].

Previous studies have studied enzyme activity or metabolites, but the cell types and localization of the different enzymes may give more relevant information. In this study, we examined the expression of two major enzymes involved in arginine depletion and replenishment in the tumor microenvironment, namely ARG2 and ASS1, respectively, in a series of non-small-cell lung carcinomas (NSCLC). Moreover, we examined the correlation of their expression with markers of intratumoral hypoxia and acidity and markers of immune response. The impact of ARG2 and ASS1 expression patterns on patients’ survival is also studied.

## Materials and methods

A series of 98 non-small cell lung cancer (NSCLC) tissue material from consecutive patients treated with surgery alone in our hospital was studied. The age of patients ranged from 32 to 82 years (median 68). Staging according to the UICC system showed the following distribution: stage I 46, stage II 22, and stage III 30 patients. Fifty-eight cases were of squamous histology, 22 cases were adenocarcinomas, and 18 undifferentiated large cell carcinomas. The median follow-up of patients was 46 months (range 26–112 months).

### ARG2 and ASS1 immunohistochemistry

For the detection of arginase-2 (ARG2), we used the primary rabbit polyclonal ab191029 antibody (abcam, UK), with 60 min incubation at dilution 1/1000. For argininosuccinate synthase ASS1, we used the goat polyclonal ab109753 (abcam, UK) with overnight incubation at dilution 1/50. The slides were deparaffinized by xylene and rehydrated in graded ethanol solutions. Microwaving was applied for epitope retrieval and using the Dako EnVision FLEX Target Retrieval Solution (pH 9.0). After washing the specimen, the slides were incubated with primary antibodies. Endogenous peroxidase was quenched with EnVision Flex Peroxidase Block (DAKO) for 10 min. Slides were subsequently incubated with the secondary antibody (EnVision Flex/HRP; DAKO) for 30 min and were washed in PBS. The color was developed by 5-min incubation with EnVsion Flex Chromogen (DAKO). Weak counterstaining with hematoxylin followed. Replacement of the primary antibody by normal rabbit immunoglobulin-G was used as a negative control. Normal liver and kidney tissues were used as positive controls.

Assessment of the expression of ARG2 and ASS1was performed at × 200 magnification. The percentage of cancer cells with strong cytoplasmic expression was recorded in all-optical fields, and the mean value was used to score each case. The extent of ARG2 and ASS1 staining exhibited by cancer-associated fibroblasts (CAFs) was recorded as the percentage of stained stroma area in the whole tissue section at × 200 magnification. Identification of cancer cells, fibroblast, and lymphocytes was based on their histological morphology.

### Assessment of other immunohistochemical markers

The membrane expression of PD-L1 by cancer cells was assessed (rabbit monoclonal anti-PD-L1 antibody, clone CAL10, Biocare Medical), as previously reported [11]. The expression of lactate dehydrogenase LDH5, Hypoxia-inducible factor 1α, and of carbonic anhydrase CA9, were assessed using the ab9002 (Abcam, UK), the ESEE122 (Abcam, UK) and the M75 antibodies (Oxford, UK), respectively, as previously described [12, 13]. The microvessel density was assessed using the pan-endothelial cell marker CD31 (ab JC70, Abcam, UK), and scoring of the microvessel density (MVD) in the invading tumor front was performed as previously reported [13].

### Scoring of tumor-infiltrating lymphocyte density

Tumor-infiltrating lymphocytes were quantified on hematoxylin-stained tissue sections, as previously described [11]. Briefly, the number of TILs infiltrating the tumor stroma only (not in the tumor nests) was assessed in all × 40 optical fields, and the mean value was obtained. Four different groups of TIL-density were defined and scored (TIL-score) as follows: 1 (minimal, mean value 1–10 lymphocytes/o.f.), 2 (low, mean value 10–70 lymphocytes/o.f.), 3 (medium, mean value 70–150 lymphocytes/o.f.), and 4 (high, mean value > 150 lymphocytes/o.f.) [11].

The iNOS expression by tumor-infiltrating lymphocytes was assessed with the primary ab15323 rabbit polyclonal antibody (abcam, UK), with overnight incubation, at dilution 1/50. The percentage of tumor-infiltrating lymphocytes (TILs) stained for iNOS (^iNOS+^TILs within total TIL presence) was also recorded in all × 40 optical fields (× 40 objective lens, × 10c eyepiece lens), and the mean score was used to characterize each case, as previously reported [14]. This percentage shows only the relative presence of such lymphocytes in the total TIL population and does not take into account the overall TIL density. Therefore, the extent of stroma infiltration by ^iNOS+^TILs was calculated by multiplying the % ^iNOS+^TILs with the TIL-score (that ranges from 1 to 4, see “Materials and methods” section). In this way, we produced the ‘^iNOS+^TIL-score’.

### Statistical analysis

The GraphPad Prism 7.0 package was used for statistical analysis and graph presentation. The chi-square or Fisher’s exact *t* test was applied to compare categorical variables, as appropriate. Linear regression analysis was used to assess the correlation between continuous variables. Kaplan-Meier survival curves were plotted to assess the impact of immunohistochemical variables on the overall survival of patients (disease-specific). Cox’s proportional hazard regression models with backward elimination were used to assess the effect of the parameters on the death events. A *p* value of < 0.05 was considered for significance.

## Results

### Expression of ARG2 and ASS1 in normal lung

**Fig. 1:**
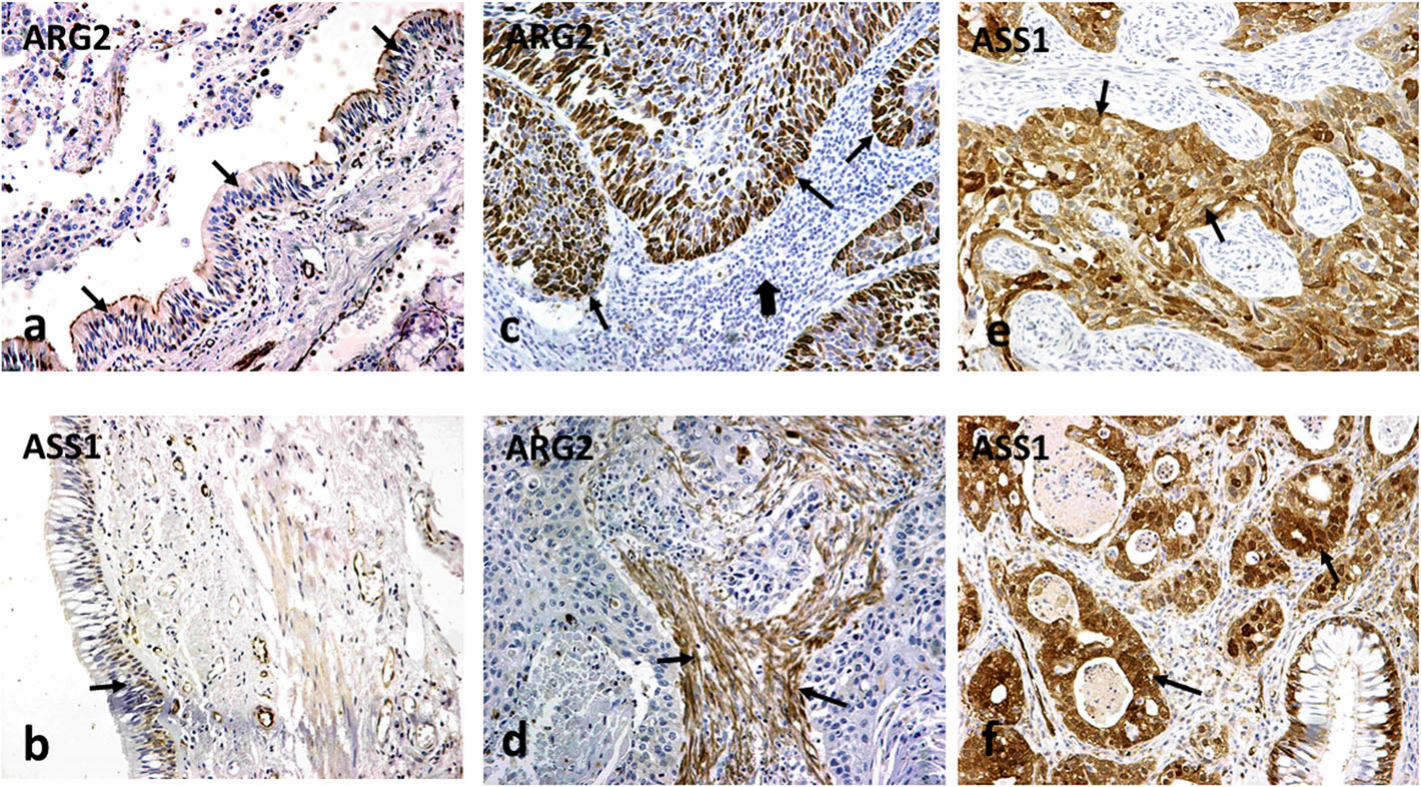
Typical immunohistochemical images of normal lung tissue showing weak expression of ARG2 and ASS1 by bronchial cells (indicated by arrows in **a** and **b**, respectively) and of lung cancer tissues (**c**–**f**). **c** A squamous lung cancer with strong ARG2 expression, while image **d** shows extensive expression of ARG2 in the tumor stroma. **e**, **f** A squamous cell cancer and adenocarcinoma, respectively, with strong expression of ASS1 by cancer cells (arrows). The thick arrow in **c** shows intense lymphocytic infiltration of the tumor stroma negative for ASS1

ARG2 and ASS1 had similar expression patterns in normal lung tissue. Bronchial and alveolar epithelium, as well as glandular epithelium, had weak expression (Fig. 1a, b). Alveolar macrophages were strongly positive for both proteins. Normal vessels sporadically expressed ARG2 and, more extensively ASS1. Fibroblasts in normal lung tissue, whether peri-bronchial, in the inter-alveolar spaces or in the stroma between the sero-mucinous glands were negative for both ARG2 and ASS1.

### Expression of ARG2 in tumors

Immunohistochemical analysis of tumor tissues showed that ARG2 is expressed in the cytoplasm of cancer cells and of cancer-associated fibroblasts (CAFs) (Fig. 1c, d). Tumor vessels occasionally expressed ARG2. Infiltrating lymphocytes did not express ARG2 (Fig. 1c).

Out of 98 cases, in 73 (72.5%) there was no ARG2 or sporadic cell expression in cancer cells. In the remaining 25 cases, ARG2 was strongly expressed in the cytoplasm of 10–90% of the cancer cell population (median 40) (Table 1).

**Table 1:** Expression patterns of ASS1 and ARG2 in tumor tissues

ASS1		
Cancer cell expression		
*Pattern*	*% cc reactivity*	*No pts (%)*
Negative-sporadic	0%	23 (23.5%)
Medium	10–40%	41 (41.8%)
High	50–100%	34 (34.7%)
Extent of stroma CAF expression		
*Pattern*	*% stroma*	*No pts (%)*
Negative-Sporadic	0%	93 (94.8)
Medium	10–40	5 (5.2)
High	50–100%	0 (0)
ARG2		
Cancer cell expression		
*Pattern*	*% cc reactivity*	*No pts (%)*
Negative-sporadic	0%	73 (74.5)
Medium	10–40%	13 (13.3)
High	50–100%	12 (12.2)
Extent of stroma CAF expression		
*Pattern*	*% stroma*	*No pts (%)*
Negative-Sporadic	0%	40 (40.8)
Medium	10–40%	31 (31.6)
High	50–100%	27 (27.6)

Regarding the stroma CAFs, no ARG2 expression was noted in 40/98 (40.8%) of cases. In the rest of the cases, ARG2 expression covered 10–90% of the stroma surface area (median 40%); Fig. 1d. In 31/98 (31.6%) ARG2 covered 10–40% of the stroma area (medium expression), and in the remaining 27/98 (27.6%) covered 50–90% of the stroma area (high expression).

**Fig. 2:**
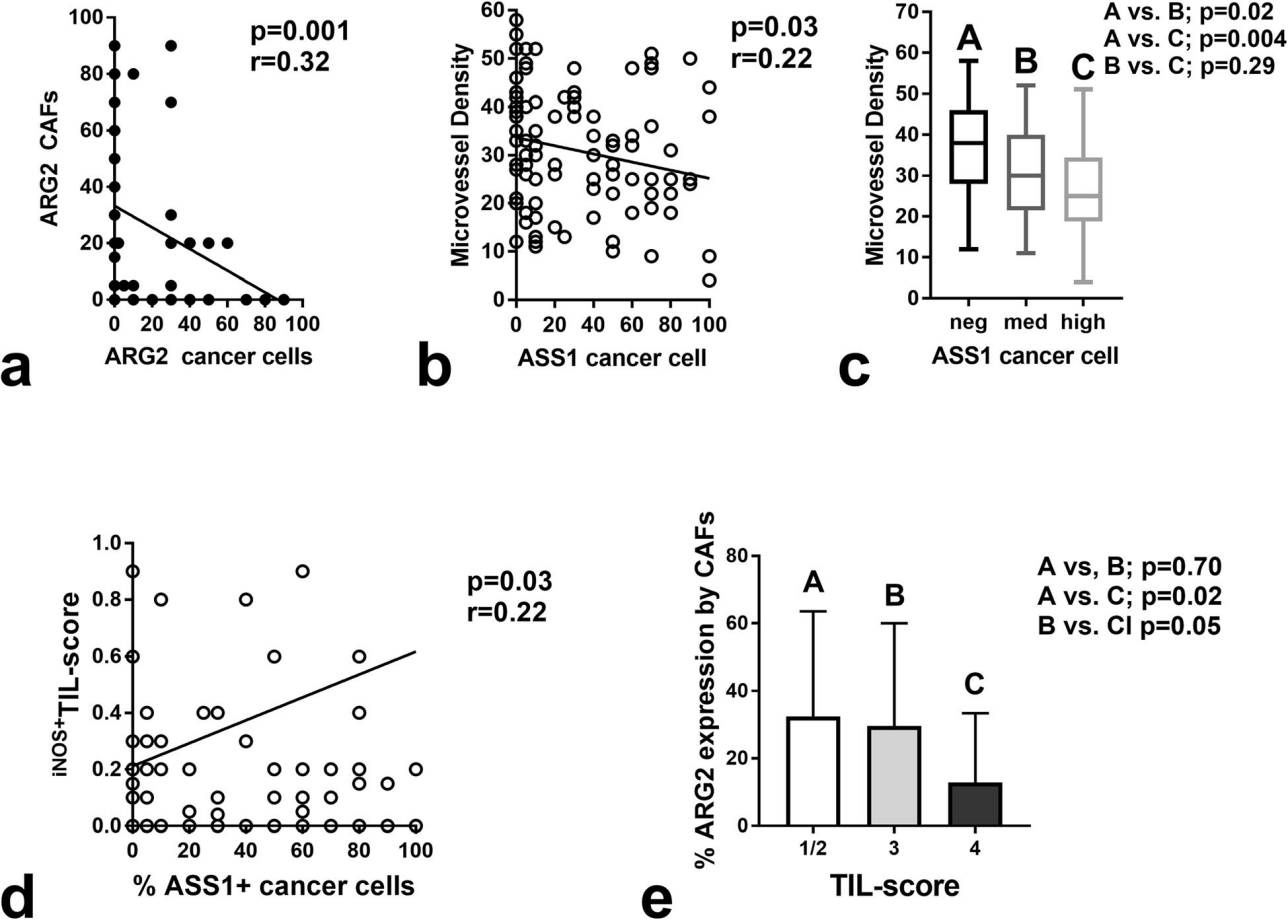
Graphic representation of important correlations. **a** Inverse association between the extent of ARG2 expression by CAFs and the % of ARG2 expressing cancer cells. **b** Linear regression analysis between the MVD and the % of ASS1 expressing cancer cells. **c** MVD according to the expression of ASS1 by cancer cells (boxes show the 25th and 75th percentiles, and bars the range). **d** Linear regression analysis of ASS1 expression by cancer cells and iNOS+ TIL-score. **e** Extend of expression of ARG2 by tumor stroma according to the TIL-score

Of interest, ARG2 expression by cancer cells and by CAFs was inversely inter-related (*p* = 0.001, *r* = 0.32**)** (Fig. 2a).

### Expression of ASS1 in tumors

Argininosuccinate synthase (ASS1) was largely expressed by cancer cells (Fig. 1e, f). Out of 98 tumor samples examined, in 23 (23.5.1%) cases, cancer cells were negative. ASS1 expression by cancer cells was noted in 75 cases (76.5%), ranging from 10 to 100% (median 40%) (Table 1).

Analysis of the extent of expression in the tumor stroma CAFs showed that no ASS1 expression occurred in the vast majority of tumors, thus 93/98 cases (negative 94.8%), while expression in 10–40% of the area of the stroma was noted in 5/98 (5.2%) cases (Table 1).

### Association between ARG2, ASS2 expression, and histopathological variables

There was no association between ARG2 and ASS1 cancer cell expression (*p* = 0.89, *r* = 0.34). ASS1was not related to the histology type or stage of the disease. A significant association of low ARG2 expression by CAFs with a low stage of disease (stage 1) was noted (*p* = 0.03) (Supplemental Table 1s).

### Correlation of ARG2 and ASS1 expression with hypoxic markers and immunological parameters

ASS1 and ARG2 expression by cancer cells or by CAFs were not related to the expression of HIF1α, LDH5, or CA9 by cancer cells. Analysis according to the microvessel density (MVD) showed that tumors that did not express ASS1 in cancer cells had a significantly higher MVD in the tumor invading front (*p* < 0.02; Fig. 2b). This was also verified in linear regression analysis, where an inverse association between the % of ASS1 expressing cancer cells and MVD was noted (*p* = 0.03, *r* = 0.22; Fig. 2c).

Neither ASS1 nor ARG2 expression by cancer cells was linked with TIL-score or PD-L1 expression. ASS1expression by cancer cells was significantly and directly related to the density of iNOS expressing TILs, the ^iNOS+^TIL-score (*p* = 0.03, *r* = 0.22**;** Fig. 2d).

ARG2 extensive expression in the stroma CAFs was inversely related with the TIL-score (*p* < 0.05); Fig. 2e).

### Survival analysis

**Fig. 3:**
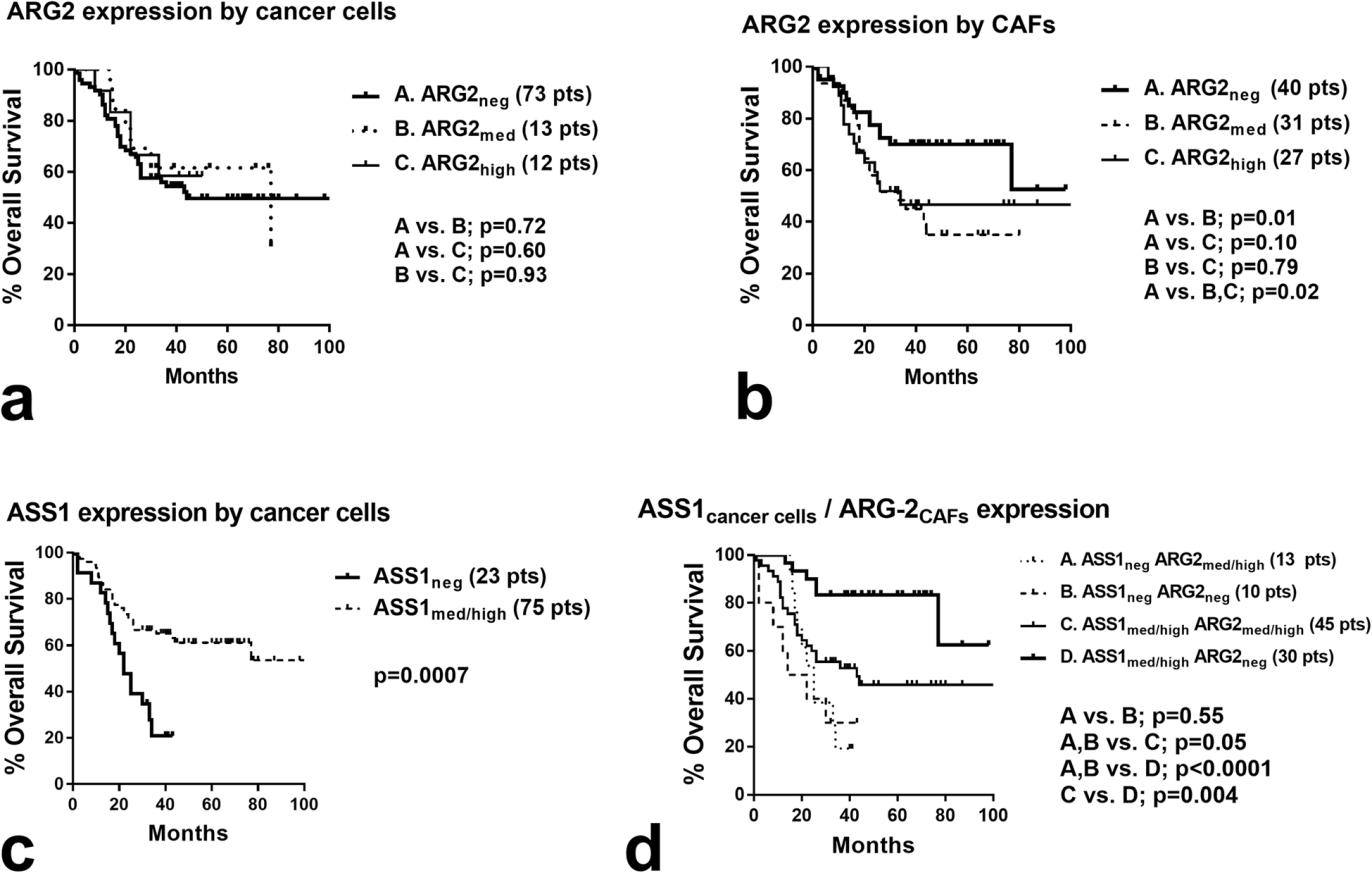
Kaplan-Meier overall disease-specific survival stratified for **a** ARG2 expression by cancer cells. **b** ARG2 expression by cancer-associated fibroblasts CAFs. **c** ASS1 expression by cancer cells. **d** Double stratification for ASS1 expression by cancer cells and ARG2 expression by CAFs

Kaplan-Meier overall disease-specific survival analysis did not show association of cancer cell ARG2 expression with prognosis (*p* > 0.60; Fig. 3a). Analysis according to the ARG2 expression by CAFs showed that extensive-expression was correlated with significantly poorer prognosis (*p* = 0.02; Fig. 3b). In a bivariate model of Cox-regression analysis, including stage and CAF-ARG2 expression, both parameters were independent predictors of prognosis (*p* = 0.001, HR 1.88[1.3–2.6] and *p* = 0.05, HR = 1.7[95% CI 0.9–3.4], respectively).

ASS1 expression by cancer cells was significantly linked with better prognosis (*p* = 0.0007; Fig. 3c). Bivariate stratification, however, showed that patients with ASS1 expression by cancer cells had a better prognosis especially when CAFs did not express ARG2 (*p* = 0.004; Fig. 3d). A multivariate analysis of stage, tumor ASS1, and CAF-ARG2 expression showed that ASS1 and stage were independent prognostic variables (*p* = 0.01, HR 0.46 [0.2–0.8] and *p* = 0.0001, HR = 3.8 [95% CI 1.9–7.8], respectively).

## Discussion

Expression of amino acid degrading enzymes in the tumor microenvironment seems to have a critical role in the anti-tumor immune response and cancer cell evasion from immune surveillance [15]. Tryptophan and arginine metabolism are the ones that have instigated the most attention of researchers. IDO (indoleamine 2,3-dioxygenase) and TDO (tryptophan 2,3 dioxygenase) convert the essential amino acid tryptophan to kynurenine, the latter having strong immunosuppressive activity and promoting tumor growth [16]. Arginine deiminases, arginases ARG1 and ARG2, as well as nitric oxide synthases NOS are enzymes metabolizing arginine, a semi-essential amino acid, necessary for the proliferation and function of T cells [7–10]. Pharmacological interference of such amino acid degrading or synthesis system may prove of therapeutic value in the treatment of cancer by facilitating immune rejection and enhancing the activity of immune checkpoint inhibitors [6, 15].

In the current study, we investigated the expression of ARG2 and ASS1 in non-small cell lung carcinomas. ARG2 was expressed by cancer cells in one-fourth of tumors examined, but this expression was not related to stage, histology, or prognosis of patients. This finding is in accordance with a previous report by Rotondo et al. [17]. In the current study, however, we documented an additional ARG2 staining pattern. ARG2 expression by cancer fibroblasts (CAFs) was a prevalent tumor feature as it concerned two-thirds of tumors. This latter pattern of expression, in contrast to cancer-cell ARG2 expression, was significantly related to an advanced stage and poor prognosis, independently of the stage of the disease.

It has been anticipated that consumption and depletion of ARG from the tumor stroma due to the overexpression of ARG2 by CAFs would be detrimental for cancer cells, which contrasts the clinical aggressiveness of tumors with high stroma ARG2 expression, as found in the current study. This finding should be, however, considered together with two important findings. ARG2 expression by stroma fibroblasts (CAFs) was related to low TIL density. Depletion of arginine in the stroma by ARG2 expression by CAFs promote, eventually, an immunologically cold tumor phenotype that explains the poor prognosis of patients with ARG2 overexpression in the stroma. Indeed, High TIL-score and high iNOS+ TIL-score (current study) relate to favorable prognosis in the current series of NSCLCs [11, 14].

The unexpected association of high CAF-ARG2 expression with a low ARG2 expression by cancer cells suggests that in this quite common tumor phenotype, cancer cells may have developed an ‘*exogenous-ARG independent’* metabolism. Overexpression, for example, of argininosuccinate synthase by cancer cells would support auxotrophy and cancer cell survival in the context of an ARG-depleted tumor microenvironment. Indeed, ASS1 was extensively expressed by cancer cells in 75% of tumor analyzed, suggesting that the majority of NSCLCs have an inherent ability to synthesize arginine. The arginine synthetic ability was, however, linked with a better prognosis. This implies that, aside from cancer cell-supporting pathways, arginine production is also useful in tumor-suppressing pathways. ASS1 expression was directly related to the high infiltration of the tumor stroma by iNOS expressing TILs, a feature being previously linked with good prognosis [14]. These findings suggest that arginine produced by cancer cells through ASS1 activity is released in the tumor stroma, allowing high arginine utilization by TILs for proliferation and activation. Moreover, ASS1 expressing cancer cells may take up less arginine from the stroma, leaving larger amounts of arginine available to TILs. High arginine consumption rates by iNOS expressing TILs predict high intratumoral NO release. NO may have important functions in T cell differentiation, cancer cell clearance through peroxynitrite production, and effector T cell activity [18–20].

This hypothesis of a link between ASS1 and enhanced immune surveillance supports the statistically significant and independent favorable prognostic value of ASS1 expression by cancer cells. Overexpression, however, of ARG2 in the tumor stroma counteracts immune surveillance since, eventually, the tumor microenvironment becomes depleted from arginine as CAFs rapidly use the amino acid. Indeed, this antagonistic interplay between ARG2-expressing CAFs and TILs resulted in poor prognosis in the current series of patients, despite the arginine synthetic activity of ASS1 expressing cancer cells. An additional explanation regarding the favorable prognostic role of ASS1 expression by lung cancer cells comes from a recent study, suggesting that ASS1 is essential to block cancer cell invasion through STAT3 pathway inhibition [21].

Auxotrophic tumors with lack of ASS1 expression by cancer cells are expected to require exogenous supplies of Arg. An auxotrophic pattern is expected to suffer from metabolic insufficiency, detrimental for the tumor. This hypothesis, however, was directly countered by the observation that auxotrophic tumors were clinically aggressive, as shown by the poor survival of patients in the current report. This was independent of ARG2 expression by CAFs. In these tumors, lack of adequate intratumoral arginine amounts is expected to block TIL anti-tumor activity. Nevertheless, other biological features may also define their aggressive clinical behavior. In the current study, we noted a direct association of auxotrophic tumors with high angiogenic activity, an ominous prognostic tumor characteristic. High levels of arginine suppress the angiogiogenic activity in experimental models [22]. Although arginine deprivation has been proposed as a sound treatment option [10], such treatment may act as a double-edge sword as it may impair immune surveillance or induce biological pathways increasing tumor aggressiveness, such as angiogenic activity.

From a therapeutic point of view, targeting the arginase pathway appears quite complex and dependent on the above-mentioned three phenotypic tumor patterns. Arginine depletion therapy with ASS1 inhibition [23] could have a role in the treatment of tumors proficient for arginine synthesis (extensive expression of ASS1 by cancer cells), provided that the tumor stroma does not already play this role by ARG2 overexpression. In this latter case, ARG2 inhibitors [24] could sustain a high intratumoral arginine content and, eventually, reverse the immunologically cold microenvironment, by allowing colonization by anti-cancer TILs. Auxotrophic tumors depend on exogenous arginine feeding, so that arginine deprivation therapy with arginine deiminase or arginase formulations [25, 26] may lead to arginine starvation and death of cancer cells. Whether anti-VEGF therapy may further assist in the eradication of auxotrophic tumors is a hypothesis that emerged from the current results (Fig. 2). There are no experimental or clinical data supporting this hypothesis. Nevertheless, arginine deiminase therapy, known for its anti-angiogenic activities [27], sensitized exclusively ASS1-deficient auxotrophic pancreatic tumors to radiotherapy through mechanisms involving ER-stress and angiogenesis suppression [28].

An important limitation of the study is that this provides a descriptive model based on immunohistochemical patterns of expression of enzymes in the tumor micorenvironment which needs validation in experimental models. Moreover, another important drawback is that functional analysis of the enzymes expressed in cancer cells and fibroblasts is certainly necessary to strengthen the ASS1/ARG2-based grouping of lung tumors. For example, acetylation of the ASS1 occurs by rhythmic interactions between ASS1 and CLOCK, leading to ASS1 inactivation [29]. Blockage of ASS1 activity may occur even under its abundant expression, and this hypothesis demands further investigation. Nevertheless, despite the oversimplification of the complex metabolic activity in tumors provided by immunohistochemical studies, distinct patterns of relative expression were identified and were significantly correlated with clinical behavior. Our data provide the spatial location and heterogeneity of metabolism and potential consequence, but is correlative. However, it provides a new basis to guide in vitro work on this metabolic pathway. The continued introduction of novel immunotherapy strategies in the clinical practice, including metabolism targeting agents, will demand biomarkers for the individualization of therapies. Immunohistochemistry has been successfully used to guide treatment individualization for estrogen receptor, HER-2 and PD-L1 expressing tumors. Whether the immunohistochemical patterns of arginine metabolism provided herein will have a similar role in arginine metabolism targeting therapies is a question that can be answered only after clinico-pathological trials.

**Fig. 4:**
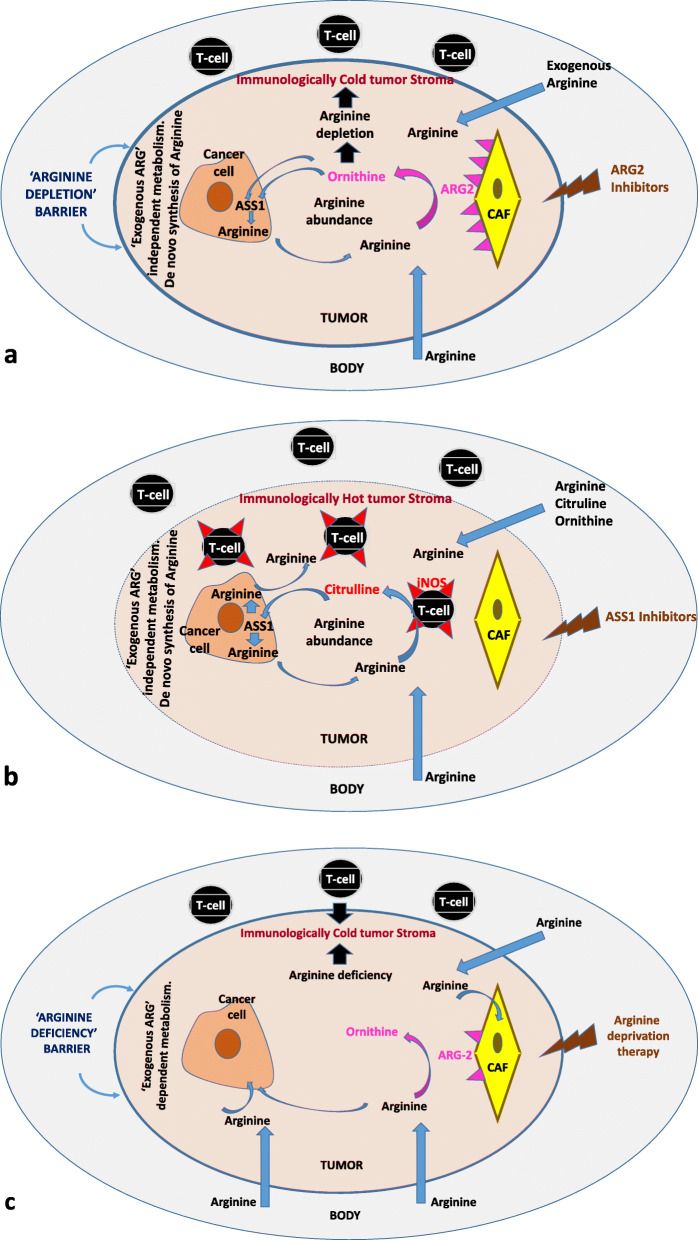
Schematic representation of the postulated biology of the three distinct NSCLC phenotypes, as emerged by the immunohistochemical findings. **a** Arginine synthesis proficient tumors expressing ASS1, with tumor stroma that does not consume arginine (ARG2 deficient). These tumors synthesize arginine for their own demands and release excess arginine to the stroma, fertilizing this to the thriving of infiltrating TILs that use arginine for their activity and proliferation. Arginine can also be used by TILs for the release of NO through arginine metabolism by iNOS enzymatic activity. As immune surveillance prevails, these tumors have a favorable prognosis. Targeting these tumors with ASS1-inhibitors may be of value provided that this will not damage the anti-tumor immune balance. **b** ASS1 expression by cancer cells in parallel with ARG2 expression by stroma CAFs predict for the depletion of the released arginine in the stroma. The low availability of arginine blocks the proliferation and activation of TILs, contributing to a cold immune environment. This is linked with a poor prognosis. Targeting these tumors with ARG-inhibitors may restore immune surveillance by fertilizing the tumor stroma for TIL thriving. **c** Auxotrophic tumors lacking ASS1 depend on exogenous arginine availability that can also be further suppressed by ARG2 expression by CAFs. These lung tumors are immunologically cold and are associated with a poor prognosis. Arginine-deprivation therapies (arginase, arginine deiminase administration) may eliminate the already low tumor arginine content, promoting cancer cell arginine starvation and death

It is concluded that ARG2 and ASS1 enzymes are extensively expressed in NSCLC. The former mainly by stroma fibroblasts and the latter by cancer cells. We identified two dominant patterns related to Arg metabolism (Fig. 4). The first comprises auxotrophic tumors with lack of ASS1 expression that has a poor prognosis. These auxotrophic tumors may protect themselves by blocking arginine-dependent TIL anti-tumor activity and exploit pathways such as angiogenesis. The second large group consists of ASS1-proficient tumors that synthesize Arg to support their growth, at the same time allowing thriving of TILs through Arg fueling and consumption by upregulated iNOS. Upregulation, however, of ARG2 in the stroma may deplete arginine and block the activity of TILs, allowing immune escape and poor prognosis. Validation of the above model to study distinct phenotypes of arginine metabolism in tumors other that lung cancer is necessary to establish a biomarker-methodology that would be useful in the individualization of arginine-targeting therapies

## Supplementary Information

**Additional file 1: Supplemental Table 1s.** Association of low ARG2 expression by CAFs with a low stage of disease.

## Data Availability

All data are available upon reasonable request.

## Abbreviations

ASS1: Argininosuccinate synthase 1
ARG2: Arginase-2
NSCLC: Non-small-cell lung cancer
iNOS: Inducible nitric oxide synthase
CAFs: Cancer-associated fibroblasts
TILs: Tumor infiltrating lymphocytes
Arg: l-arginine
