# Supplementary material for: The prognostic and therapeutic implications of distinct patterns of argininosuccinate synthase 1 (ASS1) and arginase-2 (ARG2) expression by cancer cells and tumor stroma in non-small-cell lung cancer
Source: Cancer Metab. 2021 Aug 3;9:28. doi: 10.1186/s40170-021-00264-7 (PMC8336070; doi:10.1186/s40170-021-00264-7)
Supplement: Supplementary file 1 — Additional file 1: Supplemental Table 1s. Association of low ARG2 expression by CAFs with a low stage of disease. [file 40170_2021_264_MOESM1_ESM.docx]

**Supplemental Table 1s**

**Histology**

**S A L p-value**

ARG2

*Cancer cells*

Negative (73) 42 18 13 0.75

Medium (13) 9 1 3

High (12) 7 3 2

*Stroma*

Negative (40) 25 10 5

Medium (31) 17 8 6 0.60

High (27) 16 4 7

ASS1

*Cancer cells*

Negative (23) 14 4 5 0.79

Medium (41) 26 8 7

High (34) 18 10 6

*Stroma CAFs*

Negative (93) 54 22 17

Medium (5) 4 0 1 0.45

**STAGE**

**1 2 3 p-value**

ARG2

*Cancer cells*

Negative (73) 32 17 24 0.86

Medium (13) 7 3 3

High (12) 7 2 3

*Stroma*

Negative (40) 24 6 10

Medium (31) 13 8 10 0.03*

High (27) 9 8 10

(*) stage 1 vs 2,3 – negative vs. medium/high

ASS1

*Cancer cells*

Negative (23) 8 4 11 0.31

Medium (41) 22 10 9

High (34) 16 8 10

*Stroma CAFs*

Negative (93) 44 21 28

Medium (5) 2 1 2 0.89
